# Acute Occlusion of a Persistent Sciatic Artery in a Patient with COVID-19 Infection

**DOI:** 10.3400/avd.cr.24-00126

**Published:** 2025-03-04

**Authors:** Daisuke Futagami, Taira Kobayashi, Hironobu Morimoto, Junya Kitaura, Shogo Mukai, Shinya Takahashi

**Affiliations:** 1 Cardiovascular Surgery, Fukuyama Cardiovascular Hospital, Fukuyama, Hiroshima, Japan; 2 Cardiovascular Surgery, JA Hiroshima General Hospital, Hatsukaichi, Hiroshima, Japan; 3 Cardiovascular Surgery, Ajina Tuchiya Hospital, Hatsukaichi, Hiroshima, Japan; 4 Cardiovascular Surgery, Hiroshima University Hospital, Hiroshima, Hiroshima, Japan

**Keywords:** coronavirus disease 2019 (COVID-19), persistent sciatic artery, thrombotic disease

## Abstract

Persistent sciatic artery (PSA) is an exceptionally rare vascular condition that occurs in approximately 0.025%–0.04% of the general population. We describe the case of a 51-year-old man who presented with acute left lower limb pain and high fever. He was diagnosed with COVID-19 and isolated, and conservative treatment was performed for toe pain, resulting in left toe necrosis. Computed tomography revealed PSA occlusion in the left lower extremity. We diagnosed the patient with acute occlusion of the PSA due to COVID-19. The complicated disease was successfully treated using distal artery bypass.

## Introduction

The coronavirus disease 2019 (COVID-19) pandemic continues to spread worldwide. The major risk factors for morbidity and mortality in patients with COVID-19 are advanced age and comorbidities. In critically ill COVID-19 patients, prothrombotic coagulation disorders and thromboembolisms are common complications that contribute to increased morbidity and mortality. Patients with persistent sciatic artery (PSA) are more likely to experience symptomatic local thrombosis and distal embolization. Here, we report a case of acute thrombotic PSA without aneurysm formation following COVID-19.

## Case Report

A 51-year-old man presented with sudden severe left toe pain and high-grade fever (temperature >39°C). COVID-19 was confirmed by PCR testing. Oxygen therapy was not required, and the chest X-ray showed no abnormalities. The patient was isolated for seven days. He had no significant medical history and was an active smoker. Conservative treatment for the toe pain led to necrosis of the left 5th toe. A minor amputation was performed by a previous physician. However, wound healing was not achieved. The laboratory findings were as follows: activated partial thromboplastin time (aPTT) 29.0 seconds, prothrombin time-international normalized ratio (PT-INR) 0.94, D-dimer 1.6 µg/mL, hemoglobin A1C (HbA1c) 5.3%, and blood glucose level 94 mg/dL.

Magnetic resonance angiography (MRA) was performed to diagnose vascular malformation and obstruction of the left popliteal artery, and he was admitted to our hospital. Electrocardiography (ECG) was a normal sinus rhythm. Computed tomography (CT) revealed no mobile plaques in the aortoiliac lesion.

**Figure 1:**
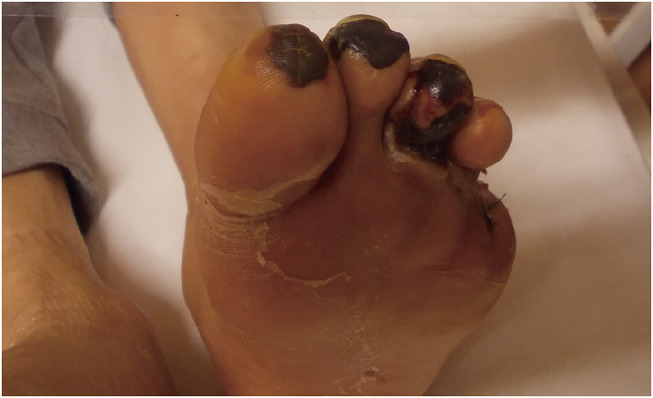
Fig. 1 Preoperative findings in the patient with partial necrosis of the first, second, and third toes and amputation of the fifth toe.

Physical examination revealed necrosis of the first, second, and third toes, with no detectable pulses in the left popliteal, dorsalis pedis, or posterior tibial arteries (PTAs; **Fig. 1**). Heparin sodium was administered intravenously and maintained at 10000 units daily, and the ankle-brachial index (ABI) was 0.74 on the left. After 14 days, conservative treatment did not lead to improvement in the necrosis.

**Figure 2:**
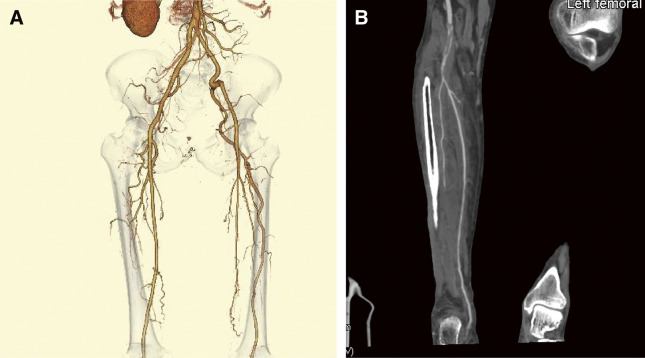
Fig. 2 CT revealed occlusion of the PSA in his left lower extremity. (**A**) The left PSA originates from the internal iliac artery, passes through the greater sciatic foramen alongside the sciatic nerve, and runs laterally to the insertion of the adductor magnus muscle before continuing as the popliteal artery. The SFA is completely absent. (**B**) The left PSA was occluded with acute thrombosis in the popliteal artery P2 segment and stenosed with atherosclerosis in the P3 segment. SFA: superficial femoral artery; CT: computed tomography; PSA: persistent sciatic artery

CT revealed left PSA, while the superficial femoral artery (SFA) was absent, and the popliteal artery (P2 segment) appeared occluded, likely due to acute thrombosis (**Figs. 2A** and **2B**). The distal popliteal artery (P3 segment) was stenosed with atherosclerosis, whereas the PTA demonstrated significant stenosis (**Figs. 2A** and **2B**).

Revascularization was performed under general anesthesia. The left inguinal region was incised to expose the origin of the SFA. A second incision was made in the mid-calf region on the left side, revealing the PTA. We performed an SFA-to-PTA bypass using a single-segment great saphenous vein graft in a non-reversed fashion. After distal anastomosis, complete angiography revealed no stenosis at the proximal or distal anastomotic sites.

The operation time was 255 min, and intraoperative bleeding was 30 mL. No peri-operative events were observed.

Aspirin (100 mg/day) was started a week before the operation, and edoxaban (30 mg/day) was administered postoperatively.

**Figure 3:**
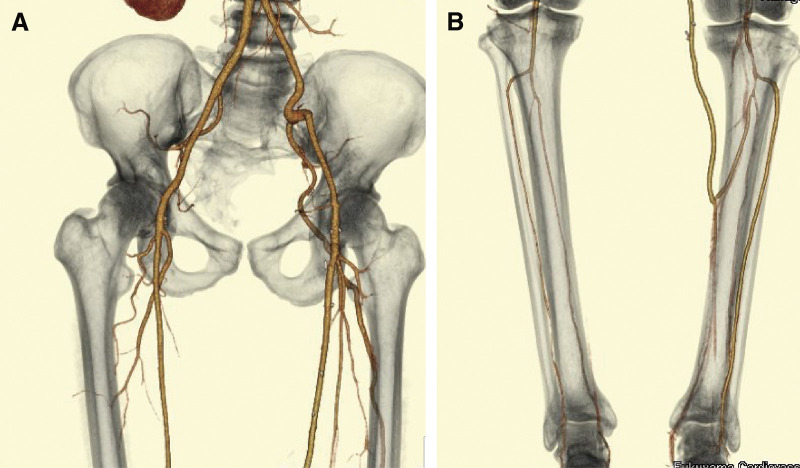
Fig. 3 Postoperative CT demonstrated that the SFA to PTA bypass is patent (**A, B**). SFA: superficial femoral artery; CT: computed tomography; PTA: posterior tibial artery

The ABI improved from 0.74 to 0.89 postoperatively, and pain in the left toe was diminished. CT demonstrated a patent SFA-to-PTA bypass (**Fig. 3**).

He was discharged on day 13 postoperative with ambulatory status.

After discharge, the necrosis of the left first, second, and third toes also improved, and no further minor amputations were needed during the follow-up period.

## Discussion

The PSA is an exceptionally rare vascular anomaly, occurring in only 0.025%–0.04% of the general population.^1)^ The PSA originates from the internal iliac artery, exits the pelvis through the greater sciatic foramen alongside the sciatic nerve, travels laterally past the insertion of the adductor magnus muscle, and then continues as the popliteal artery. There are 4 types of PSA (Supplementary Table 1).

This case had type 2b, a complete PSA with an incompletely developed femoral artery in which the SFA was absent entirely.^2)^

Thrombotic complications are common in patients with COVID-19 and tend to increase mortality and morbidity.^3–5)^ A study found that the pooled incidence of arterial thrombosis (AT) in critically ill COVID-19 patients admitted to intensive care units was 4.4%. The anatomical distribution of arterial thrombotic events was diverse, occurring in limb arteries (39%), cerebral arteries (24%), great vessels (aorta, common iliac artery, common carotid artery, and brachiocephalic trunk; 19%), coronary arteries (9%), and the superior mesenteric artery (8%). The mortality rate among these patients was approximately 20%.^6)^

The mechanisms of AT in COVID-19 patients remain unclear. Emerging evidence suggests that COVID-19 is associated with endotheliitis, characterized by extensive endothelial cell damage and infiltration by inflammatory cells.^7,8)^ This endothelial damage may result from direct viral infection, facilitated by the overexpression of angiotensin-converting enzyme 2 (ACE2), the receptor through which SARS-CoV-2 gains entry into endothelial cells.

Furthermore, COVID-19 is linked to a hypercoagulable state, characterized by elevated levels of D-dimer, prothrombin, and fibrinogen, along with shortened clot formation time and increased maximum clot firmness. This hyperviscosity is believed to stem from systemic extrapulmonary inflammation and cytokine storms, which activate the coagulation cascade. These factors—endotheliitis, hypercoagulability, and prolonged immobilization in severe COVID-19 patients—provide a plausible explanation for the development of AT.^7,9)^ The present patient was infected with COVID-19, and COVID-19 infection might have contributed to acute thrombosis in his PSA level.

Aneurysmal degeneration is seen in 41%–60% of patients with PSA, and many of these patients present with symptoms due to thrombosis, embolization, or local compression. The patient in the present case did not have an aneurysm of the PSA but developed thrombosis of the left PSA.

To the best of our knowledge, previous reports have not shown that COVID-19 causes acute thrombosis in patients with PSA.

In this patient, the PSA was not aneurysmal, but arterial sclerosis progressed, and the patient was susceptible to external forces owing to its anatomical location, which could have been a possible cause of acute occlusion during COVID-19 infection. In cases of PSA, careful observation is necessary even in the absence of aneurysms, particularly during the COVID-19 infection.

Treatment varies based on the symptoms, type of PSA, and presence or absence of an aneurysm.^2,10,11)^ In a review of 101 cases of popliteal artery aneurysm presenting with acute limb ischemia, Mann et al. noted that 46.1% (n = 47) of the patients underwent open surgical repair, 20.8% (n = 21) were treated with an endovascular approach, another 20.8% (n = 21) received a hybrid treatment, and 10.8% (n = 11) were managed non-surgically due to either mild symptoms or patient refusal of the procedure.^2)^

The report indicated that 84.3% (n = 86) of cases demonstrated good recovery over a mean follow-up period of 21 months, with 3 instances of graft or stent occlusion recorded.^2)^

This case involved type 2b PSA without SFA, and endovascular treatment was anatomically difficult to perform. In addition, the patient had severe toe necrosis; thus, we chose open bypass rather than endovascular treatment because of the increased blood supply.

## Conclusion

PSA is an extremely rare vascular phenomenon, and we encountered a patient who presented with acute thrombosis of the non-aneurysmal PSA due to COVID-19. The complicated disease was successfully treated using SFA-to-PTA bypass.

## Declarations

### Informed consent

Written informed consent was obtained from the patient for the publication of the case details.

### Ethics approval

This study was approved by the Ethics Committee of Fukuyama Cardiovascular Hospital (No. 114).

### Data availability statement

The data that support the findings of this study are available from the corresponding author, D.F., upon reasonable request.

### Acknowledgments

We would like to thank Editage (www.editage.jp) for English language editing.

### Disclosure statement

The authors declare no conflicts of interest associated with this manuscript.

### Author contributions

Study conception: DF and TK

Data collection: DF

Manuscript preparation: DF

Critical review and revision: all authors

Final approval of the article: all authors

Accountability for all aspects of the work: all authors.

## Supplementary Information

Supplementary Table 1 Type of persistent sciatic artery
