# Supplementary material for: Acute Occlusion of a Persistent Sciatic Artery in a Patient with COVID-19 Infection
Source: Ann Vasc Dis. 2025 Mar 4;18(1):24-00126. doi: 10.3400/avd.cr.24-00126 (PMC11891447; doi:10.3400/avd.cr.24-00126)
Supplement: Supplementary Table 1 — Type of persistent sciatic artery [file avd-18-1-24-00126-s01.pdf]

Supplementary Table 1 Type of persistent sciatic artery

**Type 1**	A fully developed PSA exists alongside a normally developed femoral artery.
**Type 2**	Complete PSA is present, but the femoral artery is only partially developed.
Subtype a	The SFA is present but does not extend sufficiently to form the popliteal artery.
Subtype b	The SFA is completely absent.
**Type 3**	Partial PSA where only the proximal portion remains, while the femoral arteries are fully developed.
**Type 4**	Incomplete PSA, where only the distal portion persists, with the femoral arteries developing normally otherwise.
